# A review on flexible self-healing thermoelectric devices

**DOI:** 10.1016/j.isci.2026.114701

**Published:** 2026-01-15

**Authors:** Xiaolong Sun, Yue Hou, Ziyu Wang, Yuan Yu

**Affiliations:** 1 Key Laboratory of Artificial Micro-and Nano-structures of Ministry of Education and School of Physics and Technology, Wuhan University, Wuhan 430072, China; 2 School of Integrated Circuits, Wuhan University, Wuhan 430072, China; 3 Institute of Physics (IA), RWTH Aachen University, Sommerfeldstraße 14, 52074 Aachen, Germany

**Keywords:** Physics, Engineering, Materials science

## Abstract

The rapid development of wearable and portable electronics has intensified the demand for flexible thermoelectric devices that can convert human body heat into electricity. In practical use, however, such devices suffer from performance degradation caused by mechanical damage and thermal stress, which severely limit their service life and reliability. The recent introduction of self-healing materials offers a promising route to address these challenges. This review summarizes the fundamental principles, architectures, and performance requirements of thermoelectric devices, and surveys the current research progress of self-healing materials employed therein. Evidence to date demonstrates that the rational design and optimization of self-healing materials can markedly enhance the reliability and longevity of thermoelectric devices, providing strong support for the sustainable evolution of flexible electronics.

## Graphical abstract

**Figure:**
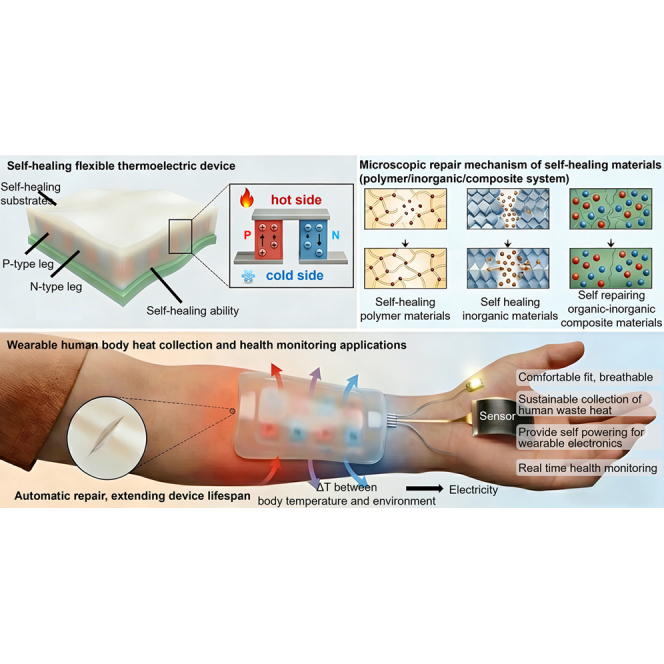

## Introduction

The digital era is witnessing an unprecedented surge in wearable and portable electronics that are reshaping how we live, work, and care for our health.^1^^,^^2^^,^^3^ From smartwatches and fitness trackers to intelligent textiles and portable medical devices, these technologies deliver unprecedented convenience and health protection. Yet, every new function, whether an extra sensor node or an always-on AI accelerator, adds to the energy budget.^4^^,^^5^ Typical power demands of today’s miniaturized wearables range from ∼100 nW for a sleep-mode Bluetooth beacon to about 10 mW for a multi-parameter health patch.^6^ Although modest in absolute terms, these values are large enough to exhaust a coin-cell battery within days or weeks, forcing users into the inconvenient cycle of recharging or replacing power cells. Bulky batteries also break the mechanical compliance expected of skin-conformable devices and raise ecological concerns over toxic waste.^7^

Notably, the human body itself is a vast energy reservoir. An adult at rest dissipates roughly 100 W of heat.^8^^,^^9^ Harvesting merely 1% of this energy as electricity would suffice to operate most wearable electronics.^7^ This untapped potential has motivated researchers to explore novel energy-harvesting strategies, among which thermoelectric (TE) devices have attracted particular attention because of their unique energy-conversion mechanism.

Thermoelectric devices enable the mutual conversion of thermal and electrical energy based on the Seebeck and Peltier effects.^10^^,^^11^^,^^12^^,^^13^^,^^14^ They can not only generate electricity from temperature gradients but also be used for active cooling, thus demonstrating unique advantages in various application scenarios. Unlike triboelectric^15^^,^^16^^,^^17^ or piezoelectric^18^^,^^19^^,^^20^ energy harvesters that rely on intermittent energy input, thermoelectric generators can provide a stable power output even during low-activity states such as sleep or sedentary periods.^21^^,^^22^^,^^23^^,^^24^ Beyond powering traditional wearable electronics, thermoelectric technology is gradually expanding into emerging fields that require both energy autonomy and mechanical reliability. Its applications have extended beyond energy harvesting to include self-powered wearable health sensors,^1^^,^^21^ body heat-powered implantable medical devices (such as cardiac pacemakers and neural stimulators),^25^^,^^26^ flexible temperature sensors for real-time body temperature monitoring,^27^ and infrared stealth systems based on active thermal management,^28^ among other high-precision sensing and thermal control functions. These diverse applications fully demonstrate the critical role of thermoelectric technology in building sustainable, maintenance-free, and intelligent energy systems as well as advanced functional devices.^7^

However, thermoelectric devices generally suffer from intrinsic brittleness^29^: traditional bismuth telluride ceramics are prone to fracture under minor strain,^30^ while organic materials are susceptible to performance degradation caused by bending, moisture, and corrosion.^31^ Microcracks can lead to the increased electrical resistance and disruption of thermal gradients, resulting in a significant degradation of output power.^32^ In critical medical applications such as cardiac pacemakers, such failures may pose life-threatening risks. Self-healing materials offer an innovative solution to this challenge.^33^^,^^34^^,^^35^^,^^36^ Inspired by biological systems, these materials can autonomously detect and repair damage, restoring both mechanical and electrical functionalities.^37^^,^^38^^,^^39^ In recent years, self-healing materials have been successfully applied in fields such as flexible circuits and stretchable displays. Integrating them into thermoelectric systems could significantly enhance the durability and reliability of devices operating in dynamic environments, while autonomous damage repair would reduce maintenance requirements, demonstrating considerable application potential.^40^^,^^41^^,^^42^

This review establishes a comprehensive knowledge framework spanning from material chemistry to device architecture, with particular emphasis on elucidating the synergistic mechanisms between advanced structural designs (such as π-type and Y-type configurations) and self-healing materials. It provides an in-depth analysis of how device geometry critically influences self-healing effectiveness. Our innovative exploration of “Lego-like” reconfigurable modular design and integrated power generation-cooling functionality within self-healing platforms presents a new paradigm for developing sustainable thermoelectric systems. Furthermore, this work prospectively identifies three fundamental challenges in the field: the inherent conflict between self-healing capability and thermoelectric efficiency, interfacial healing difficulties in multilayer device structures, and process compatibility issues in scalable manufacturing and circular recycling, thereby charting the course for next-generation self-healing thermoelectric devices.

By providing a comprehensive framework, we aim to accelerate the development of self-healing thermoelectric devices and to contribute efficient, sustainable power solutions for next-generation wearable and portable electronics.

## Basic architecture of self-healing thermoelectric devices

Thermoelectric devices operate based on the Seebeck and Peltier effects,^43^^,^^44^ enabling reversible conversion between thermal and electrical energy.^45^ A typical thermoelectric device consists of thermoelectric materials, electrodes, and encapsulation layers,^26^^,^^46^ with its performance determined by multi-physics coupling effects.^44^ Current mainstream devices largely retain the topological configuration of electrically series-connected p/n-type legs with thermally parallel paths. And thermoelectric devices are dominated by two geometric paradigms (π- and Y-type), each offering distinct advantages in heat-flow direction, stress management, and process compatibility.^44^^,^^47^

**Figure 1:**
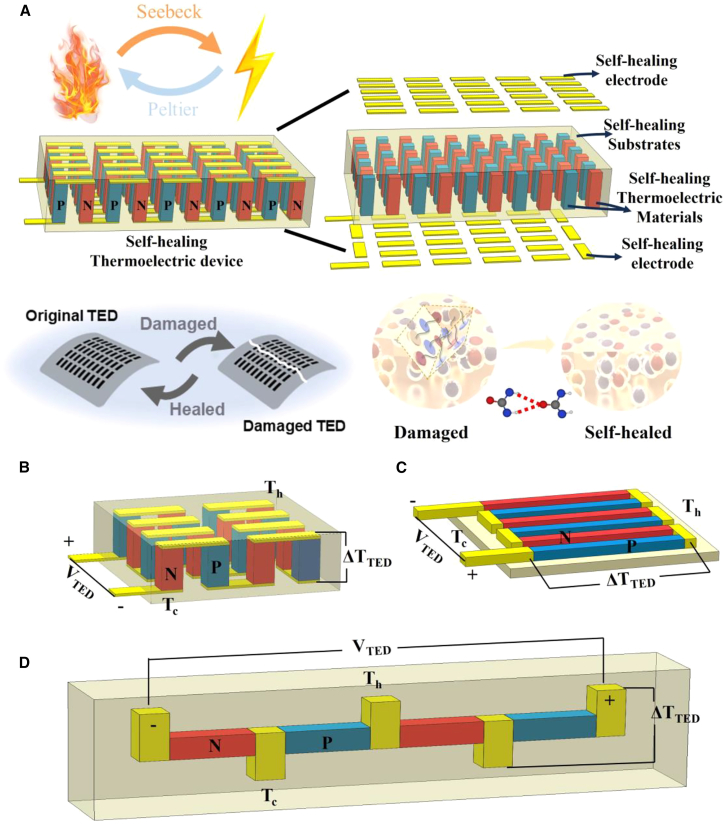
Architecture of self-healing thermoelectric devices (A) Schematic of a self-healing thermoelectric device. (B) Vertical π-type geometry. (C) Planar π-type geometry. (D) Y-type geometry.

However, prolonged operation under thermal cycling and mechanical stress can lead to failures such as electrode fracture, interface delamination, and leg cracking,^30^^,^^48^ severely limiting their application in high-reliability scenarios such as aerospace and wearable devices. To address these issues, self-healing thermoelectric devices incorporate functional materials and intelligent structures,^49^^,^^50^^,^^51^ enabling key components (thermoelectric legs, electrodes, encapsulation layers) to autonomously repair damage.^52^^,^^53^ This mechanism restores electrical, thermal, and mechanical properties, significantly enhancing device lifespan and system reliability (Figure 1A).^54^^,^^55^^,^^56^

This section will focus on the macroscopic structural design of thermoelectric devices. The detailed composition and underlying mechanisms of the self-healing encapsulation layers and thermoelectric materials will be discussed in subsequent sections. Table 1 systematically compares the structural characteristics, thermoelectric performance, and self-healing compatibility of vertical π-type, planar π-type, hybrid π-type, Y-type, and non-conventional designs, providing valuable references for device selection and optimization.

**Table 1:** Comparison of key architectures for flexible self-healing thermoelectric devices

Architecture	Advantages	Disadvantages	Typical TE performance	Self-healing compatibility
Vertical π-type^43^^,^^57^^,^^58^	1. High output power density 2. Efficient use of material’s zT 3. Well-established fabrication process	1. Poor flexibility 2. High interfacial stress under bending/thermal cycling 3. Complex integration of self-healing components	Power Density: ∼1–50 μW cm^−2^ ΔT: ∼1–20 K	Self-healing encapsulants are crucial to repair interfacial delamination and substrate cracks. Limited space for intrinsic self-healing legs due to dense, brittle material stacks.
Planar π-type^44^^,^^47^	1. High flexibility and conformability 2. Simple fabrication (e.g., printing) 3. Ease of integration with self-healing matrices	1. Low power density 2. Significant parasitic heat loss 3. In-plane ΔT is difficult to maintain	Power Density: ∼0.1–5 μW cm^−2^ ΔT: ∼1–10 K	Ideal for embedding in self-healing substrates (e.g., polymers, ionogels). Planar layout accommodates strain, facilitating the use of both extrinsic and intrinsic self-healing materials for legs and interconnects.
Hybrid π-type^59^	1. Balances power density and flexibility 2. Improved temperature gradient management	1. Complex fabrication 2. Still susceptible to lateral heat loss	Power Density: ∼5–20 μW cm^−2^ ΔT: ∼5–15 K	The hybrid structure allows for the strategic placement of self-healing materials at high-stress points (e.g., interfaces between vertical and planar sections).
Y-type^60^	1. Excellent mechanical decoupling of TE legs 2. Reduced thermomechanical stress 3. Good conformability on curved surfaces	1. Lower conversion efficiency 2. Complex electrical and thermal design 3. Significant thermal bypass	Power Density: ∼0.5–10 μW cm^−2^ ΔT: ∼5–15 K	The “sandwich” electrode structure naturally accommodates self-healing polymers as insulating fillers and encapsulants. The decoupled legs are less prone to fatal cracks, favoring long-term healing cycles.
Non-Traditional designs^61^^,^^62^^,^^63^	1. Exceptional stretchability and deformability (Origami) 2. Conformal contact with complex heat sources (Radial)	1. Niche design, often not generalizable 2. Challenging fabrication and scalability	Power Density: ∼0.1–3 μW cm^−2^ ΔT: ∼1–5 K	These architectures are often enabled by flexible/healable materials. The structural design (e.g., folds in origami) inherently mitigates stress, working synergistically with the self-healing function to recover from large deformations.

### π-type thermoelectric devices

The π-type structure has become the dominant configuration for thermoelectric modules due to its mature fabrication process, high areal power density, and ability to fully utilize the intrinsic properties of materials. Based on the relative orientation between the heat flow direction and the thermoelectric legs, it can be categorized into vertical (Figure 1B) and planar (Figure 1C) types.^57^

The vertical π-type structure sandwiches an array of p/n-type thermoelectric legs between two insulating, thermally conductive substrates, forming an electrically series-connected, thermally parallel stack via interconnecting electrodes (Figure 1B).^43^^,^^64^ This configuration features heat flow perpendicular to the substrate surface, making it ideally suited for planar heat source applications. It maximizes material utilization and enables high-density integration, while also allowing direct coupling with passive cooling systems to enhance output. However, the densely packed structure can accumulate interfacial stress due to thermal expansion coefficient mismatch, which may lead to cracking or delamination during long-term operation.

The planar π-type structure (Figure 1C) arranges thermoelectric legs parallel to the substrate, making it suitable for thin-film, fiber-based materials and fabrication processes such as printing and filtration.^47^ It offers advantages in processing flexibility and conformability to curved surfaces.^44^ However, its in-plane heat flow path is prone to parasitic thermal short-circuiting, which reduces the effective temperature difference and consequently limits output power and conversion efficiency.

The hybrid π-type combines features of both configurations: it establishes a vertical temperature gradient while arranging the thermoelectric legs horizontally, allowing heat to first penetrate the substrate vertically before being distributed laterally to each unit.^59^ This design integrates the large temperature difference advantage of vertical structures with the high integration capability of planar layouts. Nevertheless, it requires precise control of interfacial thermal resistance and advanced encapsulation techniques to suppress lateral parasitic heat flow.

### Y-type thermoelectric devices

Unlike the π-type configuration, Y-type thermoelectric devices feature an electric current flowing parallel to the heat source surface, while the heat flux remains perpendicular to the current direction (Figure 1D).^60^ In this design, p-type and n-type thermoelectric legs are alternately embedded between multilayer electrodes, forming Y-shaped heat flux channels that integrate both electrical interconnection and in-plane thermal management functions.

This configuration effectively distributes mechanical loads to the electrodes and substrate, placing the thermoelectric legs in a quasi-independent mechanical state. This mechanism alleviates thermal stress under large temperature gradients and avoids stress concentration caused by lateral arrangements. However, non-uniform temperature and current distributions within the thermoelectric legs can limit conversion efficiency. Additionally, the electrode plates bridging the hot and cold sides create significant thermal bypass, increasing optimization complexity.^44^^,^^60^ The core value of the Y-type structure lies in its conformal integration capability with complex surfaces, which is particularly critical for applications with stringent thermal management requirements such as medical implants and specialized sensors.

### Nonconventional designs of devices

Beyond the classical geometry, researchers have devised a variety of non-traditional architectures tailored to specific functions and application environments, for example, origami,^61^^,^^65^ cylindrical,^62^^,^^66^^,^^67^ and radial designs^63^^,^^68^ among them. Origami modules mount thin-film thermoelectric materials on polymer creases; the foldable substrate suppresses mechanical failure under bending or stretching.^61^^,^^65^ Cylindrical formats wrap conformally around tubular heat sources, minimizing circumferential heat loss and boosting conversion efficiency, although their performance is highly sensitive to source geometry, limiting widespread adoption.^62^^,^^66^^,^^67^ In radial devices, legs are arranged in a ring around a central hotspot; heat spreads outward, establishing a radial temperature gradient.^63^^,^^68^ This layout is uniquely advantageous for temperature sensors, thermal switches, pressure-sensitive thermal detection, or localized photo-thermal heating. Such structural innovations substantially expand the application space of thermoelectric devices and provide practical routes for wearable electronics, industrial waste-heat recovery, and micro-system energy management.

In summary, the various structural configurations of flexible TE devices, such as π-type, Y-type, and non-traditional designs, offer distinct trade-offs in heat flow management, power density, and mechanical compliance.^43^^,^^64^ However, these engineered architectures alone cannot proactively address the inevitable mechanical damage and fatigue incurred during operation.^47^ Therefore, integrating intelligent self-healing materials into these structural frameworks becomes paramount for achieving the exceptional durability and reliability required for next-generation flexible thermoelectrics.^37^ This leads us to the discussion of the next critical component: the principles and classifications of self-healing materials, which are the key to endowing these static structures with dynamic repair capabilities.^69^

## Principles of self-healing materials

Self-healing materials are a class of intelligent systems capable of autonomously detecting and repairing physical damage (such as cracks, fractures, or interfacial delamination) while simultaneously restoring their original functions (e.g., electrical/thermal conductivity and mechanical strength).^37^^,^^69^^,^^70^^,^^71^ In thermoelectric applications, these materials can be utilized in two primary forms: one category exhibits inherent thermoelectric conversion properties and can directly function as thermoelectric materials^52^; the other, although lacking significant thermoelectric characteristics, can be applied in the encapsulation and protection of thermoelectric devices.^51^^,^^53^^,^^72^^,^^73^ To date, a variety of self-healing material systems have been developed, each suitable for different application scenarios in thermoelectric devices.^52^^,^^74^^,^^75^^,^^76^^,^^77^ This chapter will first provide a brief introduction to the general classification of self-healing materials, their healing mechanisms, and their potential application prospects in the field of thermoelectrics.

### Polymer-based self-healing materials

Owing to tailorable molecular architectures, excellent mechanical compliance, and potentially low cost, polymer-based self-healing materials have emerged as one of the most promising platforms for soft electronics, wearables, and energy technologies.^78^^,^^79^^,^^80^ In contrast to inorganic systems that require elevated temperatures to activate healing, polymers can self-repair at low (<300°C) or even ambient temperatures,^69^^,^^77^^,^^81^ greatly expanding their utility in temperature-sensitive flexible devices.

**Figure 2:**
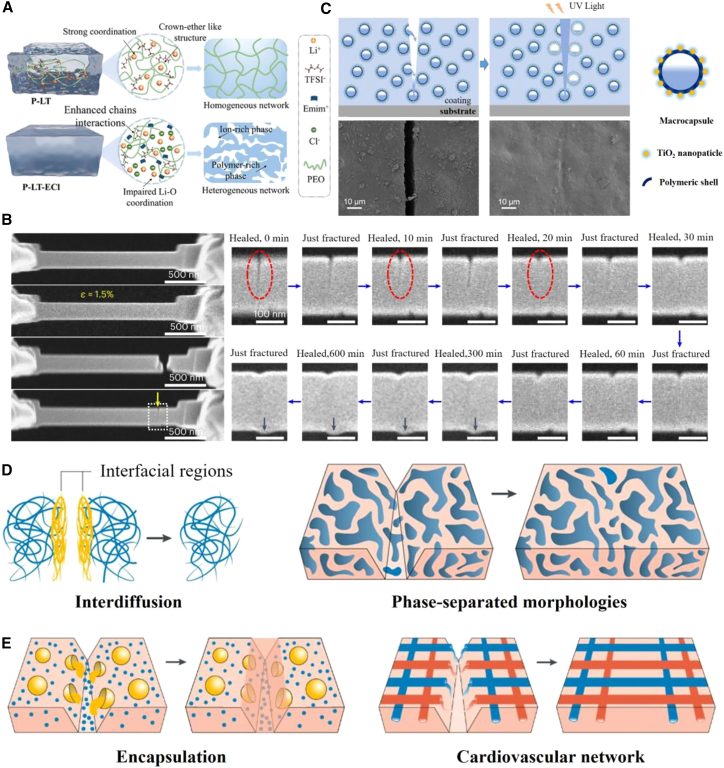
Classification and healing principles of self-healing materials (A) Polymer-based self-healing materials.^84^ (A) reproduced with permission from ref.^84^ Copyright 2024 John Wiley and Sons. (B) Inorganic-based self-healing materials.^86^ (B) reproduced with permission from ref.^86^ Copyright 2023 Springer Nature. (C) Composite/hybrid self-healing materials.^87^ (C) reproduced with permission from ref.^87^ Copyright 2019 American Chemical Society. (D) Schematic of an intrinsic self-healing system.^69^ (E) Schematic of an extrinsic self-healing system.^69^ (D and E) reproduced with permission from ref.^69^ Copyright 2020 Springer Nature.

The key advantage lies in rational molecular engineering, for example, dynamic and reversible chemical bonds or physical interactions are embedded in the polymer matrix.^69^ Upon damage, these linkages rupture and subsequently reform, “stitching” cracks and restoring properties.^82^^,^^83^ Figure 2A illustrates a representative example, which is a poly(ethylene oxide) (PEO)/lithium bis(trifluoromethanesulfonyl)imide (LiTFSI) hydrogel.^84^ Addition of the halide ionic liquid 1-ethyl-3-methylimidazolium chloride (EmimCl) introduces competitive Cl^−^–Li^+^ coordination that weakens Li-O interactions, triggering PEO chain re-conformation and *in-situ* phase separation. The resulting ternary ionogel forms a bicontinuous network: a polymer-rich phase that provides mechanical strength and stretchability, and an ion-rich phase that delivers high ionic conductivity and improved thermoelectric performance. This synergistic optimization of mechanical, self-healing, and thermoelectric properties through metal-halide supramolecular interactions highlights the versatility of polymer matrices. Collectively, these polymer-based self-healing systems provide a scalable, easily processable materials toolbox for the long-term, stable operation of flexible thermoelectric devices in complex environments.^49^^,^^85^

### Inorganic self-healing materials

Inorganic self-healing systems, i.e., based on metals, ceramics, or glasses, complement polymer matrices above ∼300°C. Healing is achieved through mechanisms such as liquid-metal flow (EGaIn, GaInSn),^88^^,^^89^ nanoparticle sintering (Ag, Cu, Ni),^90^^,^^91^ or oxidative sealing of ceramics (Al_2_O_3_, SiC, YSZ).^92^^,^^93^ For example, liquid-metal traces re-establish electrical continuity within 1 s after cracking, surviving >1000 cycles^94^; Al_2_O_3_ substrates oxidized in air at 1000°C for 2 h, forming dense α-Al_2_O_3_ nanocrystals at crack tips and recovering 80% of flexural strength, effectively arresting thermal-cycle cracks.^95^

Figure 2B illustrates the intrinsic self-healing capability of nano-twinned diamond composites (ntDC). At the fractured interface, an amorphous layer rich in sp^2^/sp^3^-hybridized carbon termed “diamond osteoblasts” (DOs) is generated.^86^ When the two fracture surfaces are brought back into proximity, the carbon atoms switch from repulsive to attractive interactions, re-establishing C-C bonds and recovering roughly 34% of the original tensile strength. The hierarchical micro-defect network within ntDC promotes abundant DO formation, whereas single-crystal diamond exhibits almost no such effect, yielding a repair efficiency below 7%.^86^ This room temperature, diffusion-free mechanism offers a fresh strategy for designing high-toughness, damage-tolerant ceramics.

Inorganic systems offer excellent thermal/electrical conductivity and corrosion resistance, yet challenges such as liquid-metal leakage, high sintering temperatures for nanoparticles, and stringent atmospheres for ceramic healing remain. Future strategies, for instance, gelling low-melting alloys, tailoring redox kinetics, and engineering gradient glass phases, will further extend their utility in high-temperature thermoelectric electrodes, ceramic substrates, and protective coatings.

### Composite-based self-healing materials

Composite self-healing systems integrate the flexibility and reversible bonding of organic polymers with the high electrical/thermal conductivity and thermal stability of metals or ceramics, creating multi-mechanism, multi-functional, wide-temperature-range cascade repair networks.^96^

Figure 2C illustrates a UV-responsive micro-capsule system engineered for aerospace coatings.^87^ The capsules use TiO_2_ nanoparticles as the outer shell and a photosensitive polymer as the inner shell to encapsulate epoxy-silicone healing agents embedded in a silicone matrix. When the coating is damaged, mechanical rupture releases the agent; simultaneously, abundant space UV radiation photocatalytically decomposes the TiO_2_ shell, triggering secondary release and crack healing. This dual-release mechanism greatly increases capsule utilization and healing efficiency, converting space UV damage into a beneficial stimulus and demonstrating strong on-orbit self-healing potential.

Through synergistic optimization of filler orientation, dynamic-bond density, and photo-thermal conversion, composite systems simultaneously achieve high stretchability (>400%), high electrical conductivity (>10^4^ S cm^−1^), high thermal conductivity (>5 W m^−1^ K^−1^), and multi-cycle healing (>50 cycles).^97^^,^^98^ They are ideally suited for flexible-rigid transition zones, multi-layer hetero-interfaces, and extreme-environment thermoelectric patches. Future research could focus on combining multiple healing mechanisms, AI-assisted multi-objective optimization, and recyclable-repairable integrated designs to boost both healing efficiency and service life of thermoelectric devices.

In Table 2, we compare three types of self-healing materials (polymer-based, inorganic, and composite materials) in terms of their healing mechanisms, healing efficiency, mechanical properties, and limitations.

**Table 2:** Comparison of self-healing materials for flexible thermoelectric applications

Material category	Healing mechanism	Healing efficiency	Mechanical properties	Key limitations
Polymer-based (e.g., Ionogels, Hydrogels)^78^^,^^79^^,^^80^	Dynamic non-covalent bonds (H-bond, ion-dipole) or Dynamic covalent bonds (imine, disulfide).	Conditions: Room Temp. - 80°C, sometimes humid air. Time: Seconds - 24 h. Efficiency: >90% (mechanical), ∼100% (conductivity).	Elongation: 100%–1000%+ Strength: 0.1–5 MPa Self-Healing Cycles: Dozens to hundreds	1. Low electrical conductivity 2. Poor environmental stability (dehydration/swelling) 3. Low thermal stability (<200°C)
Inorganic (e.g., Liquid Metal, Ceramics)^88^^,^^94^	Physical flow (LM), Oxidation-induced sealing (ceramics), Nanoparticle sintering.	Conditions: LM: Instant; Ceramics: >800°C. Time: LM: <1 s; Ceramics: Hours. Efficiency: LM: ∼100%; Ceramics: ∼80% (strength).	Elongation: LM: >500%; Ceramics: Brittle Strength: Variable Self-Healing Cycles: LM: >1000; Ceramics: Single event	1. High processing temperature (ceramics) 2. LM encapsulation challenges and weight 3. Limited mechanical strength (LM) or brittleness (ceramics)
Composite/Hybrid (e.g., Polymer filled with TE/conductive particles)^87^^,^^96^	Combined mechanisms: Polymer matrix provides healability; fillers provide TE/conductivity.	Conditions: Matrices dictate (often mild heat/light). Time: Minutes - hours. Efficiency: 70%–95% (multi-cycle).	Elongation: 50%–400% Strength: 1–50 MPa Self-Healing Cycles: Tens to hundreds	1. Complex fabrication 2. Potential filler agglomeration 3. Trade-off between filler loading (performance) and healability

### Self-healing mechanisms

To establish a quantitative link between chemical structure and macroscopic healing efficiency, a deeper understanding of the kinetics and bond energy ranges of self-healing mechanisms is crucial.^69^ The rate and efficiency of the self-healing process are fundamentally governed by the intrinsic energy and recombination kinetics of the dynamic interactions. For instance, systems relying on weak non-covalent interactions, such as hydrogen bonds (bond energy: ∼5–30 kJ/mol) and ion-dipole interactions (∼10–50 kJ/mol), often exhibit rapid (seconds to minutes) autonomous healing at room temperature, but are typically accompanied by lower mechanical strength.^69^^,^^99^^,^^100^ In contrast, systems based on dynamic covalent bonds, such as disulfide bonds (∼250 kJ/mol) and imine bonds (∼200–450 kJ/mol), provide greater mechanical robustness and more durable healing capability.^69^^,^^101^^,^^102^ However, the breaking and reformation of these bonds usually require external stimuli (e.g., heat, light) to overcome the energy barrier, resulting in healing times that can range from several minutes to hours.^69^^,^^101^^,^^102^ Consequently, the healing kinetics (from seconds to hours) and healing efficiency (often >80%, and can reach ∼100%) are directly correlated to the type, density, and spatial distribution of the dynamic bonds involved.^69^^,^^102^^,^^103^ By precisely tuning these molecular parameters, a delicate balance can be achieved between the macroscopic properties of the material (such as strength and toughness) and its ability to self-heal rapidly and efficiently, thereby providing a rational design guideline for tailoring self-healing thermoelectric materials for specific applications.^69^^,^^99^ Meanwhile, a comparison of the properties of different types of self-healing materials is also provided in Table 2.

Understanding healing mechanisms is fundamental to the design of self-healing materials. Based on the trigger mode, they can be classified as autonomous (spontaneously healing upon damage) or non-autonomous (requiring external triggers such as light or heat).^69^^,^^104^ Based on the mechanism, they are categorized as intrinsic (relying on built-in dynamic bonds within the material) or extrinsic (dependent on pre-embedded healing agents)^37^^,^^69^^,^^70^ (Figures 2D and 2E). Intrinsic healing is achieved through the reorganization of dynamic covalent/non-covalent bonds or chain diffusion,^105^ enabling multiple healing cycles without external additives. Among non-covalent interactions, hydrogen bonding, metal coordination, and ion-dipole coupling serve as common “molecular glues”^100^^,^^106^^,^^107^^,^^108^: hydrogen bonds are the most widely used due to their diverse building blocks^109^^,^^110^; metal coordination bonds offer high bond energy and directionality^111^^,^^112^; and ion-dipole interactions even enable healing in wet or underwater conditions, making them suitable for biological or marine environments.^113^^,^^114^ Non-covalent healing is essentially a reversible supramolecular assembly process: upon crack formation, dynamic groups on the fractured surfaces rapidly reassemble, driven by thermal chain motion, achieving “zipper-like” closure.^33^^,^^70^ By modulating the type of non-covalent units, binding constants, and mobility, healing temperature, speed, material modulus, and rheological behavior can be precisely programmed,^69^ enabling molecular-level restoration.

Dynamic covalent bonds (e.g., imine,^102^ disulfide,^103^ acylhydrazone^115^) possess higher bond energies, imparting greater mechanical strength and long-term healing capability to materials. However, their bond formation is relatively slow and often requires external triggers such as light or heat.^116^ Extrinsic systems rely on healing agents pre-encapsulated in microcapsules or microvascular networks,^50^^,^^117^^,^^118^^,^^119^ which are released upon damage to accomplish repair. Microcapsule-based systems offer limited healing cycles,^120^ whereas microvascular networks can store larger quantities of healing agents and enable multiple repairs,^81^^,^^104^ though their fabrication is more complex.

Self-healing materials can be integrated into thermoelectric devices in the form of encapsulation layers, electrodes, or thermoelectric media.^55^ This integration enables long-term stable operation under harsh conditions, reduces maintenance costs and replacement frequency, and thereby enhances the overall efficiency and economic viability of energy harvesting systems.

## Self-healing thermoelectric devices

Thermoelectric devices, capable of directly converting thermal energy into electricity, hold broad application prospects in energy harvesting, solid-state cooling, and wearable electronics.^4^^,^^44^ However, the inherent brittleness of conventional devices makes them prone to cracking and interfacial delamination under thermal cycling or mechanical stress, severely limiting their service life and reliability. To address this, self-healing thermoelectric devices have emerged, which autonomously restore performance after damage, significantly enhancing their durability and operational robustness.

Based on their functional roles within the device, self-healing materials can be categorized into two types: self-healing encapsulation materials^51^ and intrinsic self-healing thermoelectric materials.^84^ The former serve as protective barriers, isolating the device from environmental factors such as moisture and oxygen while sealing surface cracks induced by mechanical or thermal stress, thereby ensuring long-term module stability. The latter derive their healing capability from the material’s inherent dynamic bonding structures (including dynamic covalent bonds and non-covalent interactions), enabling autonomous recovery of electrical, thermal, and mechanical properties under appropriate stimuli.

Nevertheless, the development of self-healing thermoelectric devices still faces multiple challenges. At the encapsulation level, there exists an inherent trade-off between self-healing capability and high thermal conductivity, as the incorporation of fillers tends to restrict polymer chain mobility.^51^ The long-term reliability of interfaces between liquid metals and polymers remains questionable,^94^ and a viable economic balance between scalable manufacturing and recyclability has yet to be established.^73^ For intrinsic self-healing materials, fundamental conflicts arise between the conjugated structures or high filler loadings required for high electrical conductivity and the molecular mobility essential for self-repair.^52^ Materials such as hydrogels often suffer from inadequate environmental stability, and the activation conditions for dynamic bonds may be impractical.^84^ Additionally, the field faces systemic challenges, including complex fabrication processes and the lack of standardized testing protocols.^37^

### Self-healing encapsulation layer

For wearable applications, thermoelectric devices must simultaneously maintain high thermoelectric performance (e.g., bismuth telluride-based materials^121^) and possess flexibility and stretchability. Current mainstream solutions employ serpentine metal electrodes and elastic polymer encapsulation,^122^ yet face challenges such as high electrical resistance and susceptible solder joint fatigue. To address these limitations, liquid metal (LM) has been utilized as an electrode material due to its fluidity and low electrical resistance, demonstrating significant advantages in sustaining high output power and bending durability.^71^^,^^123^ However, the risk of leakage resulting from encapsulation failure remains a critical issue to be resolved for practical implementation.

To address the above issues, researchers have innovatively introduced self-healing materials into the encapsulation layer of thermoelectric devices and combined them with liquid-metal electrodes, successfully fabricating TEGs with self-healing capability.^51^^,^^53^^,^^72^^,^^73^ This design effectively prevents liquid-metal leakage and significantly prolongs device lifetime and reliability. Zhu et al.^53^ reported a high-performance TEG that is simultaneously recyclable, self-healing, and stretchable. The device cleverly couples commercial Bi_2_Te_3_ and Sb_2_Te_3_ legs with eutectic gallium-indium (EGaIn) interconnects and a dynamic covalent polyimine encapsulant, delivering exceptional performance and multiple advantages. Specifically, it achieves a normalized power density of 1.08 μW cm^−2^ K^−2^ and 50% mechanical stretchability, while also exhibiting outstanding self-healing and full recyclability. These features enable the TEG to recover its original performance through simple operations after damage or at end-of-life, markedly extending service life and reducing resource waste. This work offers an efficient and sustainable energy solution for future wearable electronics and highlights the broad prospects of thermoelectric technology in energy harvesting.

**Figure 3:**
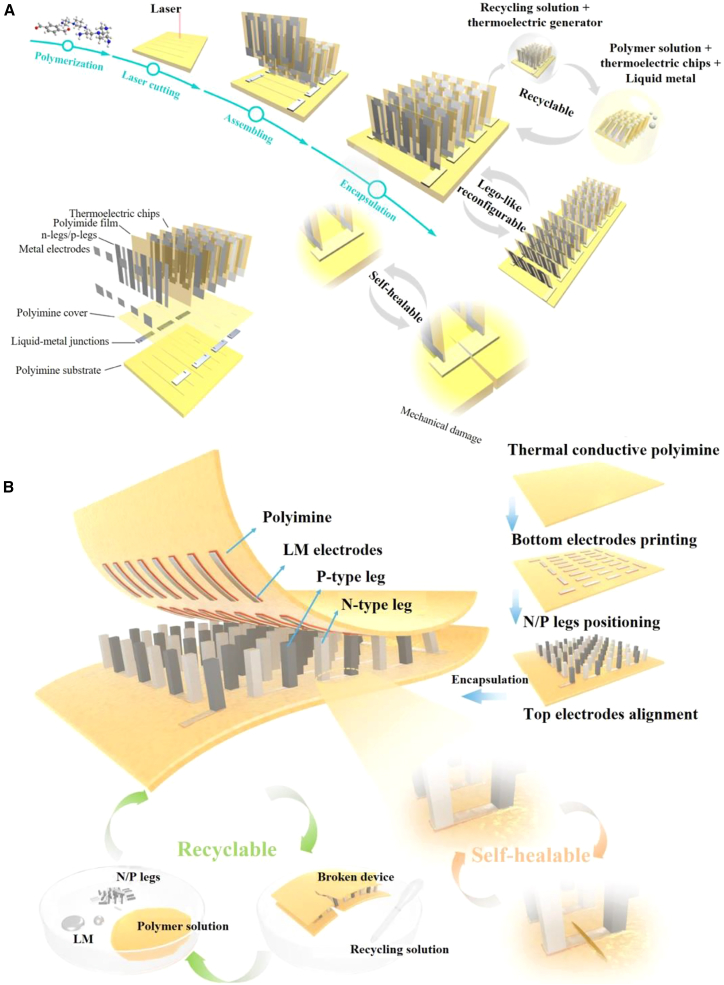
Self-healing materials as thermoelectric device encapsulants (A) Stretchable, self-healing and wearable TEG developed by Ren et al.^73^ (A) reproduced with permission from ref.^73^ Copyright 2021 The American Association for the Advancement of Science. (B) Thermoelectric device with a high-thermal-conductivity self-healing encapsulant reported by Zhu et al. for wearable energy harvesting and personal thermal management.^51^ (B) reproduced with permission from ref.^51^ Copyright 2023 Elsevier.

In parallel, Ren et al.^73^ unveiled the first high-performance wearable TEG that unites “self-healing, recyclability, and LEGO-like reconfigurability” in a single platform (Figure 3A). The authors proposed a soft motherboard and rigid plug-in (SOM-RIPs) architecture: a dynamic covalent polyimine simultaneously serves as substrate and encapsulant, while liquid metal (EGaIn) acts as the interconnect. High-ZT Bi_2_Te_2.7_Se_0.3_ and Bi_0.5_Sb_1.5_Te_3_ thin-film chips, thermally evaporated onto polyimide films, are modularly assembled into the soft matrix. Under a temperature difference of 95 K, the device delivers an open-circuit voltage density of 1 V cm^−2^ and a peak power density of 19 W cm^−2^, both of which are records for flexible TEGs. It sustains 120% tensile strain and 1,000 bending cycles at a 3.5 mm radius without performance decay. After being severed, synergistic polyimine bond exchange and LM self-welding restore LED illumination within 1.5 h; electrical continuity is maintained even at 120% strain. A 6 h chemical depolymerization-repolymerization loop enables 100% component recycling and device remanufacturing, while LEGO-like series/parallel reconfiguration is also supported. During outdoor operation, a selective absorber/emitter metasurface attached to the cold side maintains 40 mV cm^−2^ output under solar irradiation, the first demonstration of radiative sky-cooling enhancement for wearable TEGs. This work provides a readily scalable materials-process-design paradigm for next-generation stretchable, self-healing, and recyclable thermoelectric energy harvesters.

Moreover, boosting the thermal conductivity of the encapsulant significantly strengthens vertical heat exchange between the wearable TEG, the ambient, and the skin, while suppressing lateral thermal distribution. The result is a marked rise in effective power density per unit temperature difference and a corresponding amplification of output performance. Along this line, Zhu et al.^51^ incorporated high-thermal-conductivity boron nitride into a dynamic-covalent polyimine that serves simultaneously as substrate and encapsulant (Figure 3B). EGaIn liquid-metal interconnects join the legs to yield a 4.5-mm-thick, 2.7 Ω flexible module that is both recyclable and self-healing. Due to the enhanced through-plane thermal conductivity, the generator delivers a normalized power density of 1.54 μW cm^−2^ K^−2^ and, when driven with 600 mA, actively cools skin by 13.8°C (COP = 3.91). A PID feedback loop keeps skin at 32°C for 8 h or can cold-compress down to 18.2°C for fever or sprain care. For the first time, a single bulk-type TED achieves the functional integration of high-power generation, efficient active cooling, and self-healing/recyclability, providing a ready-to-deploy paradigm for wearable energy harvesting and personal thermal management in one patch.

Furthermore, Sun et al.^72^ incorporated carbon nanotubes (CNTs) into a disulfide-crosslinked polyurethane (DSPU) and employed a selective-encapsulation strategy to fabricate a self-healing, modularly assembleable flexible thermoelectric device. CNT doping raises the thermal conductivity of the healable matrix to 0.9 W m^−1^ K^−1^, yielding a record-normalized power density of 3.14 μW cm^−2^ K^−2^ for flexible self-healing TEGs. The generator maintains stable output at −50°C and 95% relative humidity, withstands 25% tensile strain and 2,000 bending cycles (r = 1.5 cm) without degradation. By LEGO-like assembly, nine TED chips are reconfigured into a large-area module. A double-decker tower architecture delivers a maximum cooling temperature difference of 6.2 K at 0.8 A, which is 60% higher than a single layer. This work pioneers the integration of selective thermal-conductivity tuning, self-healing, and modular scale-up on one flexible TED platform, offering a scalable and customizable paradigm for wearable energy harvesting and on-demand refrigeration.

**Figure 4:**
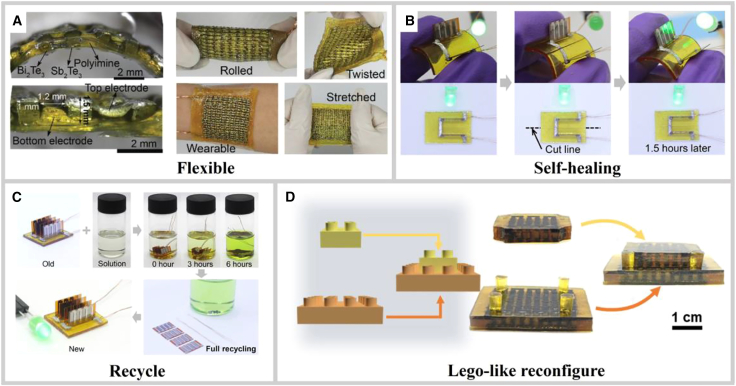
Performance of self-healing thermoelectric devices (A) Flexibility.^53^ (A) reproduced with permission from ref.^53^ Copyright 2021 John Wiley and Sons. (B) Self-healing.^73^ (C) Recyclability.^73^ (B and C) reproduced with permission from ref.^73^ Copyright 2021 The American Association for the Advancement of Science. (D) LEGO-like assembly.^72^ (D) reproduced with permission from ref.^72^ Copyright 2025 Springer Nature.

By using self-healing materials as the encapsulation layer and inorganic thermoelectric legs as the active elements, the resulting modules retain the output performance and mechanical compliance of conventional flexible TEGs while gaining self-healing, recyclability, and LEGO-like reconfigurability. As shown in Figure 4A, the generator is highly bendable, twistable, and stretchable, conforming seamlessly to complex anatomical surfaces, such as joints, wrists, or the lower back, to harvest body heat and continuously power low-power electronics such as smart bracelets and health patches.^53^ If the encapsulant is damaged during service, a gentle press at room temperature is sufficient to enable dynamic covalent bonds to reconnect and the liquid-metal traces re-establish electrical continuity, restoring original mechanical and electrical properties in an “instant-heal” fashion (Figure 4B).^73^ When the device reaches end-of-life or suffers catastrophic failure, it can be simply immersed in a recycling solution. Within minutes, the dynamic covaline network depolymerises into soluble oligomers, the liquid metal coalesces, and the thermoelectric legs and electrodes are recovered intact. Addition of aldehyde monomers to the solution, followed by re-casting, regenerates a high-integrity encapsulant matrix, while the liquid metal is washed with dilute acid, filtered, and directly reused (Figure 4C).^73^ Finally, the modular assembly protocol allows users to reconfigure multiple TE units in series, parallel, or tower stacks to create new systems tailored to different power or cooling demands, further extending the life cycle of both materials and components (Figure 4D).^72^

Although replacing traditional flexible substrates with self-healing polymers as encapsulation layers endows thermoelectric devices with unprecedented compliance, damage, repair, and recyclability functions, the thermoelectric legs still rely on high-ZT, rigid Bi_2_Te_3_/Sb_2_Te_3_ bulks. Their intrinsic brittleness (fracture strain <0.1%) and low coefficient of thermal expansion sharply contrast with the viscoelastic response of self-healing matrices. Once micro-cracks nucleate inside the legs, the electrical path is immediately interrupted, compromising long-term reliability. Moreover, the inherently low thermal conductivity of self-healing networks (typically <0.2 W m^−1^ K^−1^) severely weakens the effective temperature difference across the legs, causing a synchronous drop in output power.

Future research must therefore attack the problem on two fronts: (i) construct through-plane conductive highways with highly aligned BN, CNT, or graphene inside the self-healing encapsulant to raise thermal conductivity while preserving dynamic-bond-mediated heal-ability, and (ii) develop new deformable and healable thermoelectric systems, such as Ag_2_Se-nanosheet/CNT composite fibers, MXene-reinforced flexible Bi_2_Te_3_ thin films, or dynamically cross-linked ionogel-inorganic hybrids, that retain tensile strength >50 MPa and ZT > 0.8 under 20% tensile cycling. Only by simultaneously optimizing power output, mechanical self-healing ability, and green recyclability can the next generation of wearable TEGs be realized.

### Intrinsically self-healing thermoelectric materials

**Figure 5:**
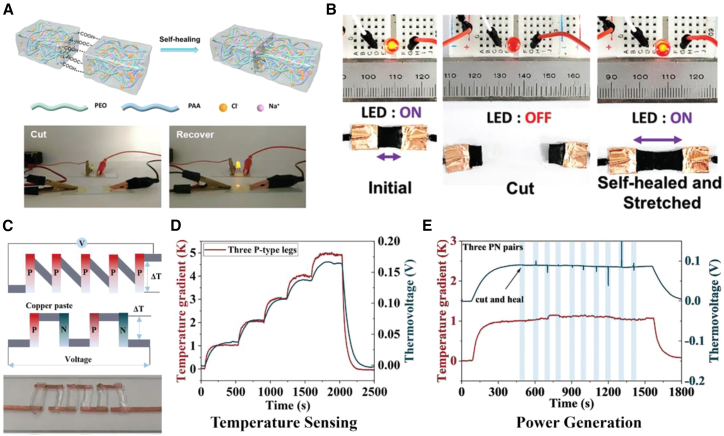
Intrinsically self-healing thermoelectric materials (A) Schematic of the self-healing process in a PAA-PEO-NaCl ionic hydrogel.^124^ (A) reproduced with permission from ref.^124^ Copyright 2023 John Wiley and Sons. (B) Photographs of an intrinsically self-healing thermoelectric material during multiple cut-heal cycles, demonstrating autonomous healing.^52^ (B) reproduced with permission from ref.^52^ Copyright 2021 John Wiley and Sons. (C) Thermoelectric device fabricated from an intrinsically self-healing thermoelectric material.^84^ (D) Intrinsically self-healing thermoelectric material used for temperature sensing.^84^ (E) Intrinsically self-healing thermoelectric material used for power generation.^84^ (C–E) reproduced with permission from ref.^84^ Copyright 2024 John Wiley and Sons.

Beyond using self-healing polymers as encapsulants, researchers have also explored intrinsically self-healing thermoelectric materials to directly extend the operational life of the legs themselves. Fu et al.^124^ reported an ionic thermoelectric hydrogel constructed from a physically cross-linked network of poly(acrylic acid) (PAA) and poly(ethylene oxide) (PEO) with NaCl (Figure 5A). The material exhibits ultra-high stretchability (>1,100%), high toughness (7.34 MJ m^−3^), and excellent self-healing capability. Reversible chain entanglement and hydrogen-bond interactions enable complete autonomous healing within 24 h at 25°C under humid conditions. Moreover, the hydrogel delivers a large ionic Seebeck coefficient of 3.26 mV K^−1^ and a low thermal conductivity of 0.321 W m^−1^ K^−1^ at room temperature, together with good adhesive properties, making it attractive for flexible ionic thermoelectric capacitors (ITECs) and other soft thermoelectric devices.

Malik et al.^52^ developed a self-healing organic-inorganic hybrid ionic-thermoelectric composite (OITC) by incorporating SiO_2_ nanoparticles into a PANI:PAAMPSA:PA ternary polymer matrix, achieving simultaneous enhancement of both ionic-thermoelectric and mechanical performance (Figure 5B). Under 80% relative humidity, the material exhibits an outstanding ionic Seebeck coefficient of 17.9 mV K^−1^ and an ionic conductivity of 0.187 S cm^−1^, yielding an ionic thermoelectric figure of merit ZTi = 3.74. This is one of the highest values reported to date. The SiO_2_ nanoparticles not only promote proton dissociation and migration but also form reversible hydrogen bonds and electrostatic interactions with the polymer chains, endowing the composite with stable performance after repeated stretching (100% strain for 50 cycles) and cutting/healing (25 cycles). Furthermore, an ionic-thermoelectric supercapacitor (ITESC) built from this material delivers an energy density of 19.4 mJ m^−2^ under a temperature difference of only 1.8 K, demonstrating excellent application potential.

Moreover, self-healing thermoelectric materials offer compelling advantages for low-grade heat harvesting and temperature sensing because of their intrinsically high Seebeck coefficients (typically in the mV K^−1^ range). Unlike conventional electronic thermoelectric materials, these ionic analogues exploit the Soret effect to generate large thermovoltages even under minute temperature differences, making them ideal for body-heat and waste-heat sources.^125^^,^^126^ Their autonomous repair capability not only extends the lifetime of both the material and the device but also enhances stability and reliability under complex mechanical stresses.

Zhao et al.^84^ reported a PEO/LiTFSI/EmimCl ternary ionogel in which metal-halogen interactions drive phase separation, overcoming the classical trade-off among self-healing ability, stretchability, mechanical strength, and ionic conductivity. By tuning the competitive coordination between Li^+^ and Cl^−^, the gel reversibly switches its thermoelectric polarity; the ionic Seebeck coefficient can be continuously adjusted from −4 to +13 mV K^−1^, and the ionic conductivity reaches 3 mS cm^−1^. As illustrated in Figure 5C, athree-leg module fabricated from this intrinsically self-healing material delivers an output voltage of 80.1 mV K^−1^, which is almost triple the single-leg value, while still responding instantly to a 1 K temperature difference. The rapid, high-amplitude signal makes the device an ultrasensitive temperature-sensing patch that can be laminated on skin or woven into wearable electronics. By constructing a reversible network of dynamic interactions, intrinsically self-healing thermoelectric materials can spontaneously, or under mild conditions, reconstruct their structure and recover their performance after mechanical damage such as cracks or fractures.

Table 3 systematically compares the healing mechanisms, healing conditions/efficiency, Seebeck coefficient, electrical conductivity, power density, and other relevant parameters for the various self-healing thermoelectric devices and materials discussed above. Among them, the first three materials are intrinsic self-healing thermoelectric materials, while the latter three serve as self-healing encapsulation materials for thermoelectric devices.

**Table 3:** Quantitative comparison of representative self-healing thermoelectric systems

Self-healing TE system	Healing mechanism	Healing conditions and efficiency	Thermoelectric properties	Output performance (device level)
PEO/LiTFSI/EmimCl Ionogel^84^	Metal-halogen coordination induced phase separation	Condition: Autonomous, Room Temperature. Time: ∼10 min Efficiency: >95%	S: −4 to +13 mV K^−1^ σ (ionic): ∼3 mS cm^−1^ κ: ∼0.3 W m^−1^ K^−1^	V_out_: ∼80 mV per leg at ΔT = 10 K Application: Tunable TE polarity, sensing
PAA-PEO-NaCl Hydrogel^124^	H-bonding, chain entanglement	Condition: 25°C, Humid Time: 24 h Efficiency: ∼100%	S (ionic): 3.26 mV K^−1^ κ: 0.321 W m^−1^ K^−1^	Application: Ionic thermoelectric capacitors (ITECs), stretchable devices
PANI:PAAMPSA:PA/SiO_2_ Composite^52^	H-bonding, electrostatic interactions	Condition: 80% RH, Autonomous Time: Minutes Efficiency: >90% after 25 cut-heal cycles	S (ionic): 17.9 mV K^−1^ σ (ionic): 0.187 S cm^−1^ ZTi: 3.74	Power: Energy density of 19.4 mJ m^−2^ at ΔT = 1.8 K
Dynamic Polyimine + Bi_2_Te_3_ Legs^53^	Dynamic covalent bonds (transamination)	Condition: 60°C, Pressure Time: ∼1.5 h Efficiency: ∼100% (Encapsulation, Electrical)	Material ZT: ∼1.0 (Bi_2_Te_3_ legs) κ_encap_: ∼0.2 W m^−1^ K^−1^	P_norm_: 1.08 μW cm^−2^ K^−2^ Feature: Recyclable, stretchable
Dynamic Polyimine + BN + Bi_2_Te_3_^51^	Dynamic covalent bonds (transamination)	Condition: 70°C, Pressure Time: ∼2 h Efficiency: >95% (Function)	Material ZT: ∼1.0 (Bi_2_Te_3_ legs) κ_encap_: ∼1.5 W m^−1^ K^−1^ (with BN)	P_norm_: 1.54 μW cm^−2^ K^−2^ Cooling: ΔT = 13.8°C
DSPU-CNT Encapsulant + TE Legs^72^	Dynamic disulfide bonds	Condition: 80°C Time: ∼1 h Efficiency: >90% (Mechanical/Barrier)	Material ZT: ∼1.2 (Bi_2_Te_3_ legs) κ_encap_: ∼0.9 W m^−1^ K^−1^ (with CNT)	P_norm_: 3.14 μW cm^−2^ K^−2^ Feature: Modular, LEGO-like assembly

While intrinsically self-healing thermoelectric materials represent a revolutionary direction toward fully autonomous and durable devices, their development faces fundamental constraints at the material level.^69^ An inherent conflict exists between the chemical structures enabling self-healing and the physical requirements for high thermoelectric efficiency: achieving high electrical conductivity necessitates high filler loading or high crystallinity, yet these features severely restrict chain mobility and dynamic bond interactions, creating a direct trade-off between conductivity and self-healing efficiency.^37^^,^^69^ Currently, the electrical conductivity of state-of-the-art self-healing conductive materials (10^2^-10^4^ S/m) remains several orders of magnitude lower than that of traditional inorganic materials.^37^ Furthermore, systems relying on dynamic non-covalent bonds are susceptible to environmental humidity, while limited thermal stability constrains their operational temperature window.^69^^,^^101^ Against this backdrop, ionic thermoelectric gels demonstrate unique value in low-grade heat harvesting and sensing applications due to their millivolt-level Seebeck coefficients and ability to restore ionic conduction pathways through reversible bonds.^84^ Meanwhile, self-healing organic/composite thermoelectric materials are striving to overcome the conductivity-healing compromise by engineering filler morphology and interfacial dynamic bonding to construct reconfigurable percolation networks that can recover after damage.^52^ In summary, the advancement of this field hinges on balancing multiple performance parameters through material innovations to propel self-healing thermoelectric technology toward practical application.^40^^,^^41^

## Conclusion

Self-healing materials enable automatic repair after damage, prevent functional failure, and dramatically extend the long-term reliability of flexible electronics, opening broad prospects for next-generation intelligent technologies. In the thermoelectric field, employing self-healing polymers as encapsulants or developing intrinsically self-healing thermoelectric materials markedly improves device robustness and lifetime.

## Application prospects

Self-healing thermoelectric materials hold great potential for various uses. In the future, we may see electronic skin that can repair itself, stick to the body to monitor health data in real time, and power itself using body heat. They could also be used to make more durable wearable devices, such as clothes or watches that can automatically heal after being scratched. In healthcare, these materials could create safe implantable devices that use body heat to work long-term without needing replacement. They could also extend the lifespan of IoT sensors, allowing them to maintain themselves in remote or harsh environments, greatly reducing electronic waste.

## Challenges and opportunities

The main challenges now are balancing how well the materials generate electricity with their ability to self-heal, while also making sure they are safe for the human body and environmentally friendly. Complex manufacturing processes and high costs are also practical issues. However, these challenges bring important opportunities: artificial intelligence can help quickly design new materials, avoiding trial and error; developing biodegradable versions could lead to truly “green” electronics; and setting unified standards can speed up the adoption of this technology. If these obstacles can be overcome, we will enter a new era and create electronics that are truly durable, maintenance-free, and eco-friendly, which would have a profound impact on building a sustainable future.

## Acknowledgments

This work is supported by the 10.13039/501100012166National Key R&D Program of China (grant no. 2023YFB4603800).

## Author contributions

X.S. wrote the original draft. Y.H., Z.W., and Y.Y. reviewed the article.

## Declaration of interests

The authors declare no competing financial interest.
